# On the Influence Exerted upon the General Temperature of the Body by a Change in the Temperature of the Extremities

**Published:** 1852-11

**Authors:** E. Brown Sequard

**Affiliations:** Paris


*On the Influence exerted upon the General Temperature of the Body by a change in the Temperature of one of the Extremities.* By E. Brown Sequard, M. D., of Paris. The following sentence is given, as an axiom, by Dr. W. F. Edwards: "We cannot either raise or lower the temperature of any one part of the body, without all the other parts of the frame being affected and suffering a corresponding rise or fall in temperature, more or less, according to circumstances."*

Expressed in such terms, we accept this law as perfectly true. But the author elsewhere gives an extension to this law, which we will prove to be incorrect. No doubt when the temperature of the blood, coming to the heart from a remote part of the body, has been modified in that part, the thoracic viscera ought to have their temperature modified; but to what extent? It is on this very important point that we do not agree with Doctor Edwards. He was of opinion that the influence exerted by a small part, on all the other parts of the body, was considerable. He says that the chilling of a single part, such as the hand or the foot, may cause a loss of temperature in all the other parts of the frame, even far beyond

*Article Animal Heat, in Todd's Cyclop. of Anat. and Physiol 1839, t. ii., p. 660.

what could have been presumed as likely or possible, and that in a number of experiments where one hand was plunged in water cooled down by ice, the other hand, which was not subjected to the action of the cold bath, lost nearly 5° R., (11°.25 F., 6°.25 Cs.,) in temperature.

It will be easily understood how important it would be that practitioners should be able to act on the general temperature of a patient, so as to increase or diminish it, by means of a hand or a foot bath. I regret to say that if the facts observed by Edwards are exact, his conclusion nevertheless is incorrect.

I have performed, both alone, and with the help of Dr. Tholozan, Physician of the Hospital *Val-de-Grace*, at Paris, numerous experiments, with the view of knowing whether Dr. Edwards was right or not. I have found that the chilling of one hand plunged in water at the temperature of freezing point, acted very strongly on the temperature of the other hand. But, at first, there is no regularity at all in the quantity of degrees of temperature lost by the hand which remains out of the water; and secondly, we have found once that this hand did not lose any fraction of its temperature. In one case we have observed that the hand kept in the atmosphere did lose 22° F., [12° Cs.] in seven minutes. The ordinary loss of temperature has been of between 6 to 8° F., [3.33 to 4°.44 Cs.] In one case there has been no loss, and, on the contrary, there has been an increase of temperature of 1°.4 F., [0°.8 Cs.]

If, like Edwards, we consider the loss of temperature of the hand not plunged in water as a sign and as a measure of the diminution of the general temperature of the body, we must conclude, from our experiments, that the chilling of a small part of the body may be unable to diminish the temperature of the body sensibly, and that, in other cases, it may act extraordinarily upon it. But the supposition that the hand kept in the atmosphere was able to give a measure of the modification of the body, is incorrect. By taking the temperature of the mouth during the time that one hand was dipped into very cold water, Dr. Tholozan and myself have ascertained that the temperature of the body does not sensibly change. The greatest diminution of the temperature of the mouth has been nearly 1° F., [0°.6 Cs.,] and this only in one case. In that experiment in which the hand not plunged in water lost 22 F., [12 Cs.] the temperature of the mouth was not diminished more than the fifth of a Fahr. degree.

As it is quite certain that the temperature of the body is constantly changing, it is easy to understand why we do not find a small but a constant diminution in the temperature of the mouth when one hand is subjected to a notable chilling. When, under the unknown influences that are constantly modifying the temperature of the body, there is a tendency to increase that temperature, then the tendency to its diminution originating from the chilling of one hand may exist without producing any effect. We have there two causes acting in opposite directions, and if they are equal they annihilate each other. If they are unequal, we perceive only the difference between them. When these two causes act in the same direction, then their efforts are added to one another, and it is probably in a circumstance of that kind that I have once found a diminution of 1° F , [0.°56 C.,] taking place in the mouth.

We have now to examine how the diminution of the temperature of a hand is produced when the other hand is dipped into cold water. *A priori* it is evident that the chilling of the hand kept in the air exists in consequence, either of the arrival of a cooler blood, or in the diminution in the quantity of blood. That hand being exposed to a cold air (for it is only in such a case that the experiments succeed,) loses its temperature by the action of that cold air. At first the blood that arrives in the hand is not cooler, as Edwards had supposed it was. This we prove by the fact that the temperature is but very little changed in the mouth. The supposition then remains that the quantity of blood arriving in the hand is smaller than usual. This may happen by two modes, one of which is that the heart sends less blood, and the other that the blood-vessels of the hand are contracted and prevent, in part, the passage of blood. It is certain that the heart continues perceptibly to send the same quantity of blood. Therefore we are induced to admit that the hand's blood-vessels are contracted. But now what is the cause of that contraction? We will try to show that it is in an action of the nervous system. Every one knows that under the influence of a sensation or of emotion, the hands, and sometimes the feet, become cold. The nervous system, in consequence of that sensation or emotion, acts upon the blood-vessels and excites them to contract. The calibre of the visible vessels is sensibly diminished. The same phenomena take place in the two hands when one is dipped into very cold water. An exceedingly violent pain is felt, the nervous centres are strongly excited, and they act then as under the influence of an emotion. Dr. Tholozan and myself have observed that the greater the pain felt, the more the temperature was diminished in the hand left in the air.

As to the influence of partial heating Dr. Edwards relates the following experiment:

"The hand being immersed in water heated to the temperature of 34° R., [108°.5 F.—42°.5 Cs.,] rose one degree of the same scale, and the temperature of other remote parts not immediately exposed to the influence of heat were found to have risen to a corresponding degree."

I have repeated this experiment of Dr. Edwards, and I have found no evident elevation in the temperature of remote parts, as the mouth and the hand, not immersed in water.

I conclude then, from the facts contained in this note, that, in general, the temperature of the body is not sensibly modified by the chilling or the heating of a small part of the frame.—*Ib.*

